# Effects of source language duration on note-decoding effectiveness in novice interpreters’ consecutive interpreting

**DOI:** 10.1007/s00426-026-02242-4

**Published:** 2026-02-12

**Authors:** Yantao Wang, Deyan Zou, Yuchen Gao, Jing Chen

**Affiliations:** 1 School of English Studies, Dalian University of Foreign Languages, West 6#, South Road, Lvshun District, Dalian, 116044 Liaoning China; 2 School of Advanced Translation and Interpretation, Dalian University of Foreign Languages, West 6#, South Road, Lvshun District, Dalian, 116044 Liaoning China

**Keywords:** Consecutive interpreting, Source language duration, Note-decoding effectiveness, Interpreter training, Novice interpreters

## Abstract

This study examines how source language (SL) duration affects note-decoding effectiveness in consecutive interpreting (CI) among novice interpreters. Grounded in Gile’s Effort Model, we used a mixed-methods within-subjects design with 30 undergraduate interpreting students who interpreted three English segments of different durations (80s, 140s, 200s). Results showed a non-linear effect of SL duration on note-decoding effectiveness, with the sharpest performance decline occurring between the 80s and 140s durations. Longer segments were associated with higher interpreting anxiety, which correlated more strongly with performance decline at extended durations. Qualitative data corroborated these findings and revealed challenges including cognitive overload and note-structure breakdown. The results provide empirical validation of Gile’s tightrope hypothesis for Phase Two of CI and offer practical implications for interpreter training and assessment, particularly regarding duration-sensitive pedagogy and standardized duration classifications in CI examinations.

## Introduction

Consecutive interpreting (CI) is a complex cognitive process requiring coordinated source language (SL) comprehension, working memory operations, and target language (TL) production. As one of the primary conference interpreting modes, CI involves rendering SL stretches ranging from a few seconds to several minutes (González, 2012; Setton & Dawrant, 2016). This study examines how SL duration relates to note-decoding effectiveness among novice interpreters, drawing on Gile’s (2009) Effort Model.

CI is commonly divided into short and long consecutive, a distinction that is particularly important in conference interpreting. Short consecutive often covers a sentence or brief idea unit and may not require notes, while long consecutive in conference interpreting can extend to 5–10 min or, per AIIC, up to about 15 min, necessitating systematic note-taking (AIIC, 2019; Cai et al., 2015; Gillies, 2017; Pöchhacker, 2022).

CI maintains its significance across diverse professional contexts, particularly in diplomatic meetings, bilateral negotiations, and small-group discussions where equipment setup is impractical or direct interaction is preferred (AIIC, 2019; Setton & Dawrant, 2016). Professional training programs and certification examinations include long consecutive segments to develop fundamental interpreting competences and prepare interpreters for high-stakes scenarios where comprehensive understanding and accurate reproduction are essential (Gillies, 2017; Pöchhacker, 2022). This approach to CI assessment reflects both its pedagogical value and its continued relevance in professional practice (Chen, 2023; Wang, 2023).

Gile’s (2009) Effort Model posits a dynamic allocation of limited cognitive resources across listening, note‑taking, memory, and production. In Phase Two (CI = Remembering + Note‑Reading + Production), the “Note‑Reading” (note‑decoding) component entails reconstructing meaning from individually generated, often elliptical notes while integrating remembered content. Building on this framework, we hypothesize that longer SL durations increase combined processing demands across remembering, note reading, and production, thereby reducing note‑decoding effectiveness among novice interpreters.

Prior research documents the difficulty of balancing comprehension and note‑taking and the resulting errors, omissions, or infelicities (EOIs) during reformulation (Gile, 2009; Seeber, 2013). Studies on parallel processing highlight CI’s multifaceted cognitive load (Dong & Li, 2020; Jin, 2010; Lin et al., 2021; Song et al., 2023; Zou & Guo, 2024). While previous studies have examined CI performance across different durations (e.g., Cai et al., 2015), no research has isolated SL duration as an independent variable while controlling for speech rate, content complexity, and individual differences to examine its specific impact on note-decoding effectiveness—a methodological gap this study addresses.

To address this gap, we adopt a mixed‑methods within‑subjects design examining how three controlled SL durations (80s, 140s, 200s—representing short, medium, and long segments typical of interpreter training contexts) affect novice interpreters’ note‑decoding effectiveness (operationalized via transformation rate: successfully decoded propositions per unit time, and transformation ratio: proportion of successfully decoded noted propositions). By focusing on SL duration as an independent variable, the study aims to inform pedagogical sequencing of input lengths and to refine theoretical accounts of cognitive load distribution in CI. Specifically, this study addresses the following research questions:

- RQ1: What is the relationship between source language duration and student interpreters’ note-decoding effectiveness in consecutive interpreting?
- RQ2: How do transformation rate and transformation ratio change as SL duration increases, and at what duration thresholds do the most significant change occur?
- RQ3: What factors are associated with or moderate the influence of source language duration on student interpreters’ note-decoding effectiveness?

## Literature review

### Theoretical framework: Gile’s Effort Model

This study adopts Gile’s (2009) Effort Model as its theoretical framework because it uniquely operationalizes note-decoding as a distinct construct within CI’s reformulation phase. Drawing on Kahneman’s (1973) capacity theory, Gile conceptualizes CI as a resource-limited activity where Phase Two is expressed as: CI = Rem + Read + P (Gile, 2009, pp. 175–176), where Rem (Remembering) recalls information not captured in notes, Read (note-Reading) decodes and extracts meaning from notes, and P (Production) reformulates TL output. The tightrope hypothesis posits that when combined effort demands exceed available resources, performance deteriorates.

For our study, longer durations increase Rem demands through two mechanisms: greater information volume requiring retention and extended temporal distance between encoding and decoding. This increased Rem load reduces available resources for Read, predicting decreased note-decoding effectiveness. We acknowledge critics argue the model oversimplifies interactive cognitive processes and may inadequately capture discourse reconstruction complexity (Mizuno, 2005; Setton, 2002). However, we maintain Gile’s framework is most appropriate because: (1) our research targets phase-specific note-decoding processes which Gile explicitly addresses, (2) alternative models (Seeber & Kerzel, 2012; Setton, 2002) address simultaneous interpreting or lack isolated note-decoding constructs, and (3) our metrics directly operationalize Gile’s Read construct.

Empirical studies support the model’s predictions. Seeber and Kerzel (2012) used pupillometry to demonstrate resource competition during high-density segments, while Korpal (2017) found omission errors correlate with increased cognitive load during complex structures. However, the model’s applicability to extended discourse remains contested. Mizuno (2005) identifies limitations in applying Gile’s framework to extended discourse, particularly regarding dynamic discourse reconstruction when working memory demands increase with longer segments, a concern directly relevant to our manipulation of SL duration (80s, 140s, 200s). Setton (2002) similarly argues the model oversimplifies interactive cognitive processes and neglects the pragmatic dimension of interpreting. Despite these limitations, Gile’s framework remains most appropriate for this study because it directly addresses the phase-specific constraints we investigate. Our operationalization of transformation rate and ratio directly measures the Read construct, allowing us to test whether increased Rem demands from longer durations compromise Read capacity, a phase-specific prediction that alternative models do not address.

This framework informs our operationalization: transformation rate (successfully decoded propositions per unit time) measures Read efficiency; transformation ratio (proportion of successfully decoded noted propositions) measures Read effectiveness. By manipulating SL duration (80s, 140s, 200s) while controlling speech rate, content complexity, and individual differences, we test whether increased Rem demands reduce Read resources, testing Gile’s tightrope hypothesis for Phase Two note-decoding.

### Empirical studies on cognitive demands and temporal factors in CI

#### Cognitive load and resource competition

Yan et al. (2024) demonstrated that CI imposes significantly higher mental demand compared to simultaneous interpreting and other interpreting modes, with Phase Two reformulation requiring intensive coordination of memory retrieval and language production. Similarly, Chen and Dong (2025) found that articulation processes play an important role in cognitive control during language production, suggesting that the reformulation phase—which involves articulation of TL output—represents a key locus of resource competition in bilingual processing. Zhao et al. (2022) found that working memory capacity significantly predicts CI performance, particularly for tasks requiring sustained information retention and transformation. Meta-analytic evidence further confirms that professional interpreters demonstrate greater working memory capacity than bilingual non-interpreters and monolingual controls (Mellinger & Hanson, 2019), particularly in verbal-based tasks (Ghiselli, 2022). Notably, such working memory advantages emerge through interpreting experience, with beginner interpreters showing no significant advantage over bilingual controls, while intermediate and expert interpreters demonstrate considerable gains in both working memory and short-term memory spans (Wen & Dong, 2019).

#### Note-taking and note-decoding as distinct processes

Research establishes note-taking and note-decoding as distinct cognitive operations with differential resource demands. Notes function as memory reinforcers rather than transcription tools (Ahrens & Orlando, 2022; Dam, 2021; Liu et al., 2023), with Zhou and Dong (2024) demonstrating that note-taking significantly enhances both accuracy and fluency of source text recall, with facilitative effects increasing as expertise develops. Dam (2021) found expert interpreters produce more TL notes, suggesting simultaneous SL processing and TL preparation. Zou and Guo (2024) demonstrated that interpreters optimize note-taking strategies for dual-task coordination through deliberate practice.

However, note-decoding involves distinct cognitive demands beyond note-taking: information retrieval from notes, synthesis of noted and memorized content, and rapid language reformulation (Gillies, 2017). This process is particularly susceptible to temporal pressures, as interpreters must simultaneously access memory traces, decode notes, and produce TL output within strict time constraints (Gile, 2009). Recent evidence from bilingual language control research (Chen & Dong, 2025) demonstrates that articulation processes—the overt production of language output—play a more important role in cognitive resource allocation than pre-articulatory formulation processes, suggesting that in CI, where reformulation involves articulation of TL output, the articulation phase may represent a bottleneck in note-mediated CI performance. Mellinger (2023) characterizes notes as integral components of distributed cognitive processing that actively contribute to meaning construction. Despite the recognized importance of note-decoding, little research has examined how specific features of notes—such as duration of information—influence this integration process under temporal pressure.

#### Duration and temporal factors: an underexplored domain

While extensive research has addressed note-taking strategies and overall CI performance (Dam, 2021; Gillies, 2017; Zou & Guo, 2024), studies examining how SL duration affects note-decoding remain limited. Existing research acknowledges duration as a difficulty factor—Cai et al. (2015) identify segment length as contributing to task complexity—but no studies manipulate duration while controlling speech rate, content complexity, and individual differences, to isolate temporal effects on Phase Two performance. (Research demonstrates that under time pressure and extended durations, note-structure breakdown becomes a challenge (Zhou & Dong, 2024), suggesting that duration-specific note-taking strategies may be necessary for effective note-decoding.

The choice of language in which notes are taken remains an open debate and may interact with duration effects. While some advocate a-lingual approaches to minimize cognitive load (Gillies, 2017), others argue noting inherently involves linguistic processing (Dam, 2021). Liu et al. (2024) found that increased cognitive load reduces language switching in Chinese-English bilinguals, suggesting potential benefits of single-language systems during complex tasks, though individual differences significantly influence strategies (Korpal & Mellinger, 2025). Psychological factors including anxiety also affect performance: Chiang (2010) demonstrated that anxiety negatively impacts linguistic proficiency and interpreting quality, while Hubscher-Davidson (2020) emphasized stress management as key for sustained performance. However, no research examines whether anxiety interacts with duration to compound note-decoding difficulties.

### Research gap and present study

The literature reveals an important gap: no controlled studies isolate duration effects on note-decoding while systematically controlling speech rate, content complexity, and individual differences. Prior research conflates duration with content variables (longer speeches typically contain more complex content), preventing causal inference about temporal effects. While Gile’s model predicts resource competition, empirical validation for duration-specific effects on Phase Two is absent. Understanding whether performance deterioration follows linear or threshold patterns has direct pedagogical implications for training sequencing, yet this remains unexplored. Additionally, the roles of anxiety, note quality, and individual differences in moderating temporal impacts require investigation.

The present study addresses this gap through a mixed-methods within-subjects experimental design manipulating SL duration (80s, 140s, 200s) while controlling speech rate, content complexity, and individual differences. Following Zou and Guo (2024) and Chen (2017, 2022), we operationalize note-decoding through transformation rate (successfully decoded propositions per unit time, measuring efficiency) and transformation ratio (proportion of successfully decoded noted propositions, measuring effectiveness). Propositions are identified following Goldman-Eisler’s (1972) classification of predicate-argument structures, expanded to include single content words functioning as independent meaning units to accommodate student interpreter competence levels.

This design directly tests Gile’s tightrope hypothesis for Phase Two in CI: whether increased Rem demands from longer durations reduce available resources for Read processing, manifesting as decreased transformation rate and ratio. The findings will provide empirical foundation for duration-progressive training protocols and standardized assessment practices in interpreter training.

## Research design

This study employs a mixed-methods within-subjects experimental design to examine how SL duration affects note-decoding effectiveness in CI, following established best practices in cognitive interpreting research (Rojo López & Muñoz Martín, 2025). The within-subjects design enables direct comparison of each participant’s performance across three different SL durations (80s, 140s, 200s) while controlling for speech rate, content complexity, and individual differences. This section describes the participants, research instruments, materials, procedure, and data collection and analysis.

### Participants

The study involved 30 junior students (29 females, 1 male; mean age = 21 years, SD = 0.8) majoring in translation and interpreting. All participants were native Chinese speakers with no professional interpreting experience beyond academic training. They had completed CI courses in the previous semester, accumulating one semester of systematic note-taking training (approximately 36 contact hours). Selection criteria ensured participants possessed sufficient English listening proficiency and CI skills. All participants had achieved minimum IELTS scores of 7.0 (mean = 7.3, SD = 0.4), and their CI course performance ranged between 80 and 85 out of 100 (mean = 82.5, SD = 1.8), assessed using standardized criteria including accuracy, fluency, and completeness.

To ensure scientific rigor, stratified random sampling was employed. Participants were first stratified into three grade bands based on their previous semester’s interpreting course performance (80.0-81.9，82.0-83.9，84.0-85.0), with 10 participants randomly selected from each stratum to ensure sample representativeness across skill levels. Participants were then randomly assigned to different experimental time slots to control for temporal effects. Participants were recruited through announcements in relevant university courses. The study was approved by the University’s Institutional Review Board, and all participants provided written informed consent before participating in the study.

Research indicates that interpreters’ individual differences—including personality traits, emotional stability, and stress management capabilities—may significantly impact performance (Korpal & Mellinger, 2025). By controlling for learning experience, CI performance levels, and English proficiency, this study minimized these factors as potential confounds. While the sample exhibits gender imbalance (96.7% female), this demographic characteristic reflects the typical gender distribution in interpreting programs and professional interpreting contexts (Korpal & Mellinger, 2025; Magnifico & Defrancq, 2020). Gender was not a variable of interest in this study; the research focused on the relationship between SL duration and note-decoding effectiveness. The homogeneity in participants’ academic background, skill level, and linguistic profile provides a solid foundation for investigating these research questions.

### Research instruments

This study employed three primary research instruments to collect both quantitative and qualitative data: CI tasks, retrospective interviews, and expert evaluations. This multi-instrument approach enabled comprehensive examination of the relationship between SL duration and note-decoding performance, with quantitative measures addressing performance outcomes and qualitative data providing supplementary insights into underlying cognitive processes.

#### CI tasks

Three CI tasks of varying durations (80s, 140s, 200s) were designed, covering stress reduction techniques. These tasks provided quantitative measures of interpreting performance across different SL durations.

#### Retrospective interviews

Semi-structured interviews were conducted immediately after the interpreting tasks, focusing on participants’ cognitive processes, strategies, and perceived challenges. These interviews yielded qualitative insights into participants’ interpreting experiences.

#### Expert evaluations

Two professional interpreters with over 10 years of experience evaluated the interpreting performances using a standardized rubric that assessed successfully interpreted propositions.

### Materials

The study employs a CI task comprising three segments of varying durations, utilizing a 996-word IELTS mini lecture as source material. The direction of interpreting is from English to Chinese (L2 to L1), chosen to focus on syntactic conversion processes while minimizing confounding variables (Xu & Liu, 2024). Three segment durations (80s, 140s, 200s) were selected based on theoretical and practical considerations, representing short, medium, and long CI segments commonly encountered in interpreter training (Gillies, 2017; Pöchhacker, 2022) that remain appropriate for novice interpreters.

The L2-to-L1 direction was selected for several methodological reasons. While L1-to-L2 interpreting might reduce listening comprehension issues, variations in participants’ L2 production abilities could obscure the focus on syntactic conversion. Although L2-to-L1 interpreting may increase cognitive demands on trainee interpreters (Chou et al., 2021), it enables more consistent output in participants’ native language and avoids the complexities of quantifying L2 production levels.

Several factors were considered in material preparation: delivery speed, subject familiarity, and readability. Research on interpreting has identified 95–120 words per minute (wpm) as optimal for simultaneous interpreting (Gerver, 1976; Pöchhacker, 2022), with speech rates exceeding 140 wpm considered problematic due to increased cognitive demands (Barghout et al., 2015; Gile, 2009). Although these findings primarily concern simultaneous interpreting, rapid delivery similarly affects CI by compromising both the Listening and Analysis Effort during note-taking and the Production Effort during reformulation (Gile, 2009). To ensure a manageable speech rate for student interpreters, the audio was modified using nvplayer.exe. Specifically, the audio was decelerated by 20%, extending duration from 7 to 8.5 min and reducing speech rate from 142 to 117 wpm, thereby mitigating the negative impact of rapid speech on cognitive load (Gile, 2009).

Pre-test results confirmed that the lecture topic (stress reduction techniques) did not significantly impact interpreting performance across participants, indicating that topic familiarity and prior knowledge were controlled as potential confounds. The material’s readability, assessed using Flesch’s (1948) formula, yields a score of 71.6, classified as “standard” difficulty, ensuring that the content itself does not introduce undue challenges.

The material was divided into three segments with durations of approximately 1, 2, and 3 min, presented sequentially from shorter to longer. This structure enabled isolation of SL duration effects on note-decoding effectiveness. Participants received standardized instructions and prompting cards before the video-recorded interpreting sessions to ensure consistent task administration.

### Procedure

The study was conducted in two phases: a pilot study and the main study. The pilot study ran for two weeks, allowing for refinement of research tools and methods, followed by a six-week main study period. This timeline ensured adequate participant recruitment and data collection while maintaining study relevance.

Three undergraduate students with profiles similar to the target participants were recruited from the university’s translation and interpreting department based on academic performance and language proficiency. The pilot procedure was conducted in a language lab equipped with computers and writing materials and included brief preparation, task introduction, CI of three audio segments, and semi-structured interviews. The entire process was video-recorded to capture both note-taking and interpreting output. Based on pilot feedback, material adjustments were made, including the addition of prompting cards with main ideas and challenging vocabulary, and removal of individual long sentences that significantly impacted comprehension. These modifications resulted in three segments of 80, 140, and 200 s, representing incrementallyshort, medium, and long CI segments based on established training practices (AIIC, 2019; Dam, 2021), while maintaining consistent delivery speed (117 wpm).

Thirty participants completed the formal experiment in the same lab setting. The procedure comprised familiarization with prompting cards, CI tasks with audio segments presented from shortest to longest (80s→140s→200s), unrestricted interpretation time after each segment, researcher marking of misinterpretations and omissions, participant marking of unrecognizable notes, and cued retrospective interviews. The fixed ascending order was chosen to minimize initial anxiety by starting with manageable durations and reflect authentic pedagogical progression in interpreter training. While this design does not fully control for order effects, we acknowledge that performance decline may be partially attributable to cumulative fatigue, which is addressed through qualitative analysis of participant reports (as detailed in the qualitative analysis). Each session lasted approximately 30–45 min.

All interpretations were independently scored by two raters, achieving strong inter-rater reliability (Cohen’s kappa = 0.85, exceeding the minimum acceptable value of 0.80). Data analysis involved quantitative measures (proposition recall rates) and qualitative analysis of interview responses. The study received Institutional Review Board approval, with all participants providing written informed consent and being informed of their withdrawal rights. Confidentiality was ensured through unique identifier codes, with all data stored securely on encrypted devices accessible only to the research team. Post-experiment debriefing sessions allowed participants to ask questions or raise concerns.

### Data collection and analysis

All interpretations were audio-recorded and transcribed for analysis. Two expert evaluators independently scored the interpretations using predetermined criteria, achieving strong inter-rater reliability (Cohen’s kappa = 0.85). Semi-structured interviews were conducted immediately after each interpreting task to capture participants’ reflections on their note-taking strategies and perceived challenges.

The quantitative analysis focused on two primary measures: transformation rate (successfully decoded propositions per unit time) and transformation ratio (proportion of successfully decoded noted propositions) across the three SL duration conditions (80s, 140s, 200s). One-way repeated measures ANOVA was conducted to examine the effect of SL duration on interpreting performance (α = 0.05), with descriptive statistics computed to identify performance patterns across conditions.

Interview data underwent thematic analysis through coding to identify patterns in note-taking strategies and challenges across different SL durations. Interpretation errors and strategies observed in the transcripts were similarly coded and analyzed. Expert evaluators’ comments were analyzed to identify common challenges and effective strategies associated with each duration condition.

This multi-instrument mixed-methods design with supplementary qualitative data enabled comprehensive examination of both the quantitative impact of SL duration on interpreting performance and the qualitative aspects of how interpreters manage varying input lengths.

## Results

### Source language duration and note-decoding effectiveness (RQ1)

To examine the relationship between SL duration and CI performance, we employed a one-way repeated measures ANOVA to analyze the impact of SL duration on student interpreters’ note-decoding effectiveness. Data were gathered from the CI task across three different durations (80s, 140s, 200s) (Table 1).

**Table 1:** Results of one-way repeated measures ANOVA for interpreting performance

Source	df	MS	F	*p*	partial η²
OP	2, 58	156.42	22.23	< 0.001	0.28
MO	2, 58	148.76	22.33	< 0.001	0.29

OP = Overall Performance; MO = Ratio of Misinterpreting and Omissions

A one-way repeated measures ANOVA (Table 1) showed significant effects of SL duration on both student interpreters’ overall performance (F(2, 58) = 22.23, *p* < .001, ηp² = 0.28) and the ratio of misinterpretations and omissions (F(2, 58) = 22.33, *p* < .001, ηp² = 0.29). According to Cohen’s (1988) benchmarks, these effect sizes (ηp² = 0.28-0.29) represent large effects, indicating that duration differences have practical significance for interpreting performance, with approximately 28–29% of the variance in these measures attributable to SL duration alone. To determine which specific durations had significant influences, we conducted post-hoc analyses using Tukey’s Honestly Significant Difference (HSD) test to control for Type I error across multiple comparisons. The results are presented in Table 2.

**Table 2:** Multiple comparisons using tukey’s HSD test for OP and MO

Panel A: Overall Performance (OP)					
Duration Pairs	Mean Difference	SE	p	95% CI	
				Lower	Upper
80–140 s	0.12	0.024	< 0.001	0.08	0.17
80–200 s	0.23	0.026	< 0.001	0.18	0.28
140–200 s	0.10	0.028	0.003	0.05	0.16
**Panel B: Ratio of Misinterpreting and Omissions (MO)**					
Duration Pairs	Mean Difference	SE	p	95% CI	
				Lower	Upper
80–140 s	0.13	0.025	< 0.001	0.09	0.18
80–200 s	0.24	0.027	< 0.001	0.19	0.29
140–200 s	0.10	0.026	0.002	0.05	0.16

Positive mean differences indicate better performance for shorter durations

The post-hoc analysis reveals significant differences between all pairs of durations for both measures. Notably, the significance levels for comparisons between 80s and longer durations (*p* < .001) were more pronounced than between 140s and 200s (*p* = .003 for OP and *p* = .002 for MO), suggesting a non-linear performance decline with a steeper drop from 80s to 140s. These findings suggest that SL duration significantly impacts note-decoding effectiveness, with performance deteriorating as input length increases, particularly between short (80s) and medium (140s) durations.

### Transformation rate and ratio patterns (RQ2)

**Fig. 1:**
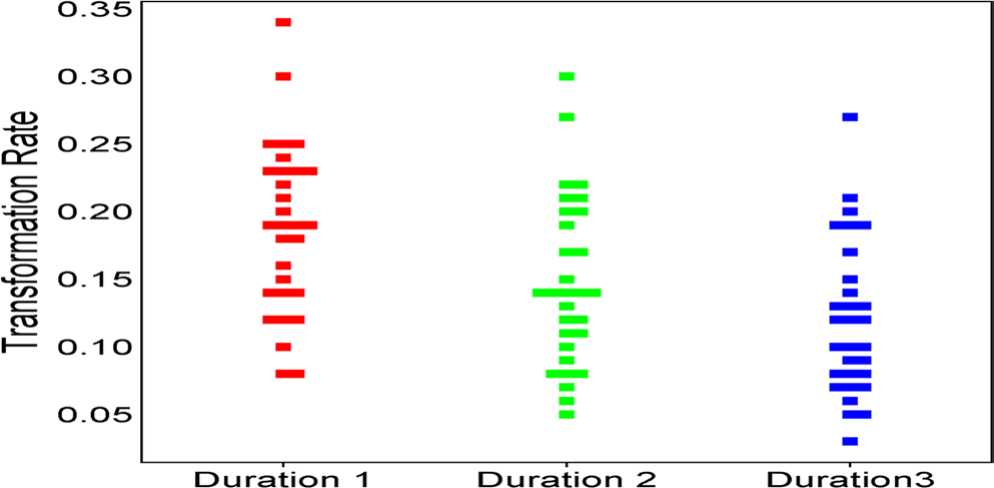
Mean transformation rate by source language duration. Note. Points represent mean transformation rates at each duration level (80s, 140s, 200s). Error bars represent ± 1 SE (*N* = 30)

**Fig. 2:**
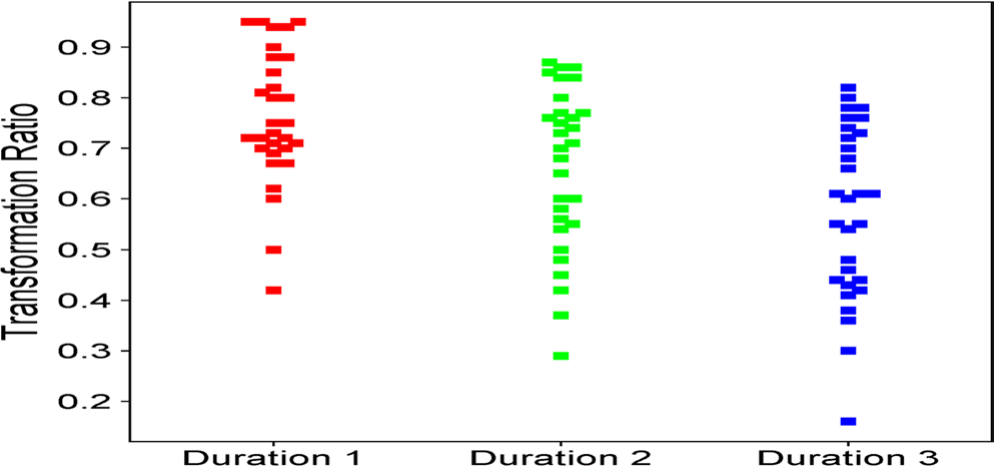
Mean transformation ratio by source language duration. Note. Points represent mean transformation ratios at each duration level (80s, 140s, 200s). Error bars represent ± 1 SE (*N* = 30). All pairwise comparisons were statistically significant (*p* < .01)

To examine the patterns of note-decoding efficiency across different SL durations, we analyzed transformation rates and transformation ratios for all 30 participants. The former was calculated as the number of successfully decoded propositions divided by total time (propositions per second), while the latter represented the proportion of noted propositions that were successfully decoded. Figures 1 and 2 present the mean transformation rates and ratios across the three duration conditions. Both measures showed consistent declining patterns as SL duration increased. Mean transformation rates decreased from 0.28 propositions per second at 80s to 0.16 at 140s and 0.12 at 200s, representing a 57% overall decline. The steepest decline occurred between 80s and 140s (43% decrease, *p* < .001), while the decline between 140s and 200s was more moderate (25% decrease, *p* = .003), confirming a non-linear pattern.

A one-way repeated measures ANOVA confirmed that these differences were statistically significant (F(2, 58) = 9.61, *p* < .001, ηp² = 0.24). Post-hoc comparisons using Tukey’s HSD test revealed significant differences between all duration pairs: 80–140 s (Mean Difference = 0.12367, *p* < .001), 80–200 s (Mean Difference = 0.22700, *p* < .001), and 140–200 s (Mean Difference = 0.10333, *p* = .003). The larger effect size between 80s and 140s compared to 140s and 200s reflects a non-linear decline pattern.

Individual performance analysis revealed both consistency and variability in response to increased duration. Twenty-six participants (86.7%) demonstrated monotonic decline across all three durations, while four participants (13.3%) showed irregular patterns with performance fluctuations. These four participants, identified as “resilient performers” in subsequent analysis, exhibited distinct cognitive strategies that warrant further investigation. The range of transformation rates narrowed progressively: 0.08–0.34 at 80s (range = 0.26), 0.05–0.30 at 140s (range = 0.25), and 0.03–0.27 at 200s (range = 0.24). Notably, three participants (10% of the sample) consistently maintained performance in the top decile across all conditions, with transformation rates of 0.30–0.34 at 80s, 0.26–0.30 at 140s, and 0.23–0.27 at 200s, representing performance approximately 2–3 standard deviations above the group mean. While these high-performing participants also experienced performance decline as duration increased (mean decline of 23% from 80s to 200s), their rate of decline was notably less steep than the overall group average (57% decline), suggesting potential individual differences in cognitive resilience or note-taking strategies.

**Fig. 3:**
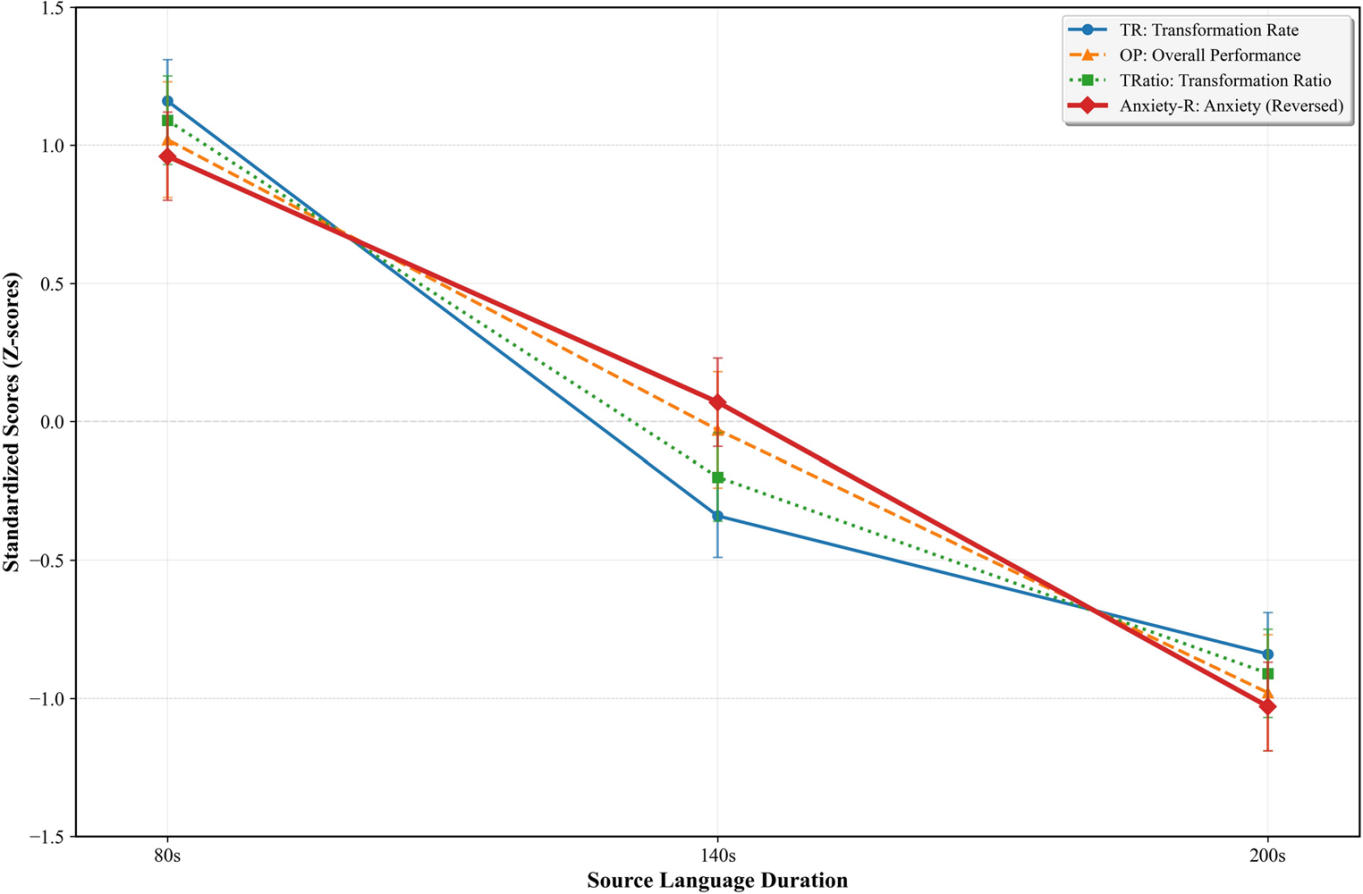
Comprehensive effects of source language duration on key performance and psychological measures. Note. This figure simultaneously displays four indicators: transformation rate (TR), overall performance (OP), transformation ratio (TRatio), and self-reported anxiety. TR, OP, and TRatio are presented as standardized z-scores; Anxiety is shown as raw scores (1–5). Error bars indicate ± 1 SE

Figure 3 presents a comprehensive view of how multiple key measures respond to duration increases. The standardized presentation reveals several important patterns: (1) all performance measures show similar declining trajectories with the steepest drops between 80s and 140s, (2) anxiety follows an inverse pattern with accelerating decreases at longer durations (indicating increasing anxiety levels), and (3) the convergent pattern across performance measures suggests a common underlying mechanism driving duration effects. Notably, the anxiety trajectory shows the steepest increase between 140s and 200s (M = 3.1 to M = 4.1, Cohen’s d = 1.34), while performance measures show their steepest decline between 80s and 140s (Cohen’s d = 1.15–1.24), suggesting that cognitive overload precedes peak anxiety responses. This temporal dissociation suggests that performance deterioration may trigger subsequent anxiety increases rather than anxiety directly causing performance decline.

These findings suggest that cognitive overload, rather than anxiety, serves as the primary mechanism driving performance decline with increased SL duration, with anxiety emerging as a secondary response to cognitive strain.

### Mediating factors in duration effects (RQ3)

To identify factors potentially mediating the relationship between SL duration and note-decoding effectiveness, we examined participants’ self-reported anxiety levels, perceived difficulty, and segment length assessments through retrospective interviews.

#### Anxiety and performance relationship

Correlation analyses revealed significant negative relationships between self-reported anxiety levels and performance scores across all three durations. As duration increased, both anxiety levels rose and the strength of the anxiety-performance correlation intensified: 80s (*r* = − .45, *p* < .01), 140s (*r* = − .58, *p* < .001), and 200s (*r* = − .67, *p* < .001), aligning with Fig. 3’s observed delay in peak anxiety response (Table 3).

**Table 3:** Correlations between anxiety and performance across durations

Duration	Anxiety		Performance		Correlation		
	M	SD	M	SD	*r*	*p*	95% CI
80s	2.3	0.8	0.75	0.12	− 0.45	0.012	[-0.68, − 0.12]
140s	3.1	0.9	0.63	0.15	− 0.58	< 0.001	[-0.77, − 0.29]
200s	4.1	1.0	0.52	0.18	− 0.67	< 0.001	[-0.82, − 0.42]

Anxiety was measured using a single-item 5-point Likert scale administered immediately after each task (“How anxious did you feel during this interpreting task?” 1 = Not anxious at all, 5 = Extremely anxious). Performance scores represent overall performance (correctly interpreted propositions / total source propositions). Higher anxiety scores indicate greater self-reported anxiety levels. All correlations significant at *p* < .05

A repeated measures ANOVA confirmed significant main effects of duration on both anxiety levels (F(2, 58) = 15.30, *p* < .001, ηp² = 0.35) and performance scores (F(2, 58) = 10.70, *p* < .001, ηp² = 0.27). Post-hoc comparisons using Tukey’s HSD test indicated that anxiety levels increased significantly between each duration pair (all *p* < .05). The cumulative increase from 80s to 200s was notable (Mean Difference = 1.8, SE = 0.22, *p* < .001), with the largest single-step increase occurring between 140s and 200s (Mean Difference = 1.0, SE = 0.18, *p* < .001).

#### Individual variations in anxiety-performance patterns

While the overall sample demonstrated significant anxiety increases and performance declines with longer durations, individual response patterns varied. Visual inspection of anxiety-performance trajectories and descriptive grouping based on median splits of anxiety change scores (80s to 200s) revealed three distinct response patterns. The largest group, “High-anxiety decliners” (*n* = 18, 60%), showed anxiety increases exceeding 1.5 points on the 5-point scale and performance drops greater than 0.20 in overall performance. “Moderate responders” (*n* = 8, 26.7%) exhibited anxiety increases between 0.8 and 1.5 points and performance declines of 0.10–0.20. “Resilient performers” (*n* = 4, 13.3%) maintained anxiety increases below 0.8 points despite duration increases, corresponding to the irregular performance patterns noted in the Results section.

#### Perceived difficulty and segment length

Table 4 presents the distribution of participants’ feedback regarding perceived difficulty and segment length appropriateness across the three durations. For the 80s duration, the vast majority rated the segment as “Simple” (82.1%), with perceptions of segment length relatively balanced between “Short” (40.0%), “OK” (36.7%), and “A little long” (23.3%). For the 140s duration, while 60.0% still rated difficulty as “Simple,” retrospective interviews revealed that 8 participants found this segment more challenging than 80s, despite their formal ratings. This discrepancy suggests that immediate difficulty ratings may not fully capture experienced cognitive load. Regarding length, perceptions shifted dramatically, with 86.7% rating it as “OK.” For the 200s duration (*n* = 27, excluding three “not clear” responses), 51.9% rated difficulty as “Simple,” yet interviews revealed that 10 participants experienced increased difficulty. For segment length, 56.7% rated it “a little long,” indicating that over half found the duration challenging.

**Table 4:** Distribution of participants’ feedback across duration

Duration	Perceived Difficulty (*n* = 30)			Perceived Segment (*n* = 30)			
	A little difficult	Standard	Simple	Short	OK	A little long	Long
80s	1 (3.6%)	4 (14.3%)	23 (82.1%)	12 (40.0%)	11 (36.7%)	7 (23.3%)	0 (0%)
140s	4 (13.3%)	8 (26.7%)	18 (60.0%)	0 (0%)	26 (86.7%)	4 (13.3%)	0 (0%)
200s	3 (11.1%)	10 (37.0%)	14 (51.9%)	0 (0%)	13 (43.3%)	17 (56.7%)	0 (0%)

For perceived difficulty: 80s (*n* = 28), 140s (*n* = 30), 200s (*n* = 27). For perceived segment length: all durations (*n* = 30). Eight participants indicated 140s was more difficult than 80s. Ten participants indicated 200s was more difficult than previous segments. Three participants indicated “not clear” for 200s difficulty. Percentages calculated based on valid responses

Chi-square tests of independence were conducted to examine the association between duration and participants’ perceptions. For perceived difficulty, the analysis included 85 valid responses (28 at 80s, 30 at 140s, and 27 at 200s, excluding “not clear” responses and non-responses), revealing a significant association (χ²(4, *N* = 85) = 18.45, *p* = .001). For segment length appropriateness, the analysis included all 90 responses (30 per duration), showing a significant association (χ²(4, *N* = 90) = 52.30, *p* < .001). All expected cell frequencies exceeded the minimum threshold of 5, satisfying the assumptions for chi-square analysis.

The divergence between formal difficulty ratings and retrospective interview responses warrants attention. While formal ratings showed relatively stable or even decreasing difficulty perceptions (with 51.9% of valid responses rating 200s as “Simple”), interview data revealed that notable proportions of participants (8 for 140s, 10 for 200s) experienced increased difficulty. This pattern suggests that participants may have employed different evaluative criteria when providing formal ratings versus describing their experiences retrospectively, or that the formal rating scale may not have adequately captured the multidimensional nature of task difficulty in CI.

#### Qualitative patterns from retrospective interviews

Thematic analysis of post-task interviews (*N* = 30) following Braun and Clarke’s (2006) framework identified three primary themes related to duration effects. To describe the prevalence of each theme, we conducted frequency analysis by counting the number of participants who explicitly mentioned each theme in their interviews.

First, anxiety and nervousness were explicitly mentioned by 23 participants (76.7%) when discussing longer durations, with representative comments including “I am very nervous… from beginning, I feel stressful” (Participant 9) and “when you tell me that this duration is over 3 minutes, I feel… nervous and stressful” (Participant 7). These anxiety-related responses aligned with the quantitative anxiety data presented in Table 3, providing qualitative validation of the measured anxiety increases.

Second, cognitive overload symptoms were reported by 19 participants (63.3%), such as “my mind even is empty in seconds” (Participant 22) or “I just want to give up and don’t want to take notes anymore” (Participant 21). These descriptions suggest that longer durations may exceed participants’ working memory capacity, leading to breakdown in note-taking and decoding processes.

Third, adaptation challenges were described by 12 participants (40%), with some noting improved strategies over time while others experienced cumulative fatigue. For instance, Participant 15 stated: “At first I tried to write everything, but by the third part I realized I needed to be more selective.” Conversely, Participant 18 reported: “Each segment felt harder than the last because I was already tired from the previous ones.”

These qualitative findings provide rich contextual understanding of the quantitative patterns observed in performance decline, anxiety increases, and individual variation in response to duration increases.

## Discussion

This study examined how source language (SL) duration affects note-decoding effectiveness in novice interpreters’ consecutive interpreting (CI) while controlling for speech rate and topic complexity. Across three training-relevant durations (80s, 140s, 200s), we observed declines in transformation rate and ratio, with the steepest performance drop occurring between short and medium segments. These non-linear patterns, corroborated by increased anxiety levels and qualitative evidence of cognitive overload, refine our understanding of Phase Two processing in CI and offer pedagogical implications for interpreter training.

### Theoretical implications: validating and refining Gile’s model

Our findings provide empirical support for Gile’s (2009) tightrope hypothesis during CI’s reformulation stage (Phase Two: CI = Rem + Read + P). By operationalizing “Read” effort as note-decoding effectiveness (transformation ratio) and efficiency (transformation rate), we found that longer SL durations increase Remembering (Rem) demands through two mechanisms: greater information volumes and extended temporal distance between encoding and decoding. This increased Rem load constrains available cognitive resources for both Reading (note-decoding) and Production (TL reformulation), manifesting as measurable performance decline, a pattern consistent with neuroscientific evidence showing that increased interpreting demands trigger heightened brain activation that impairs performance efficiency (Yan et al., 2024) and disrupt predictive processing strategies that normally facilitate comprehension (Zhao et al., 2022).

The notable effect sizes—28% of performance variance (ηp² = 0.28) and 29% of error variance (ηp² = 0.29) attributable to duration alone—establish SL duration as an important determinant of interpreting outcomes. This aligns with previous research showing that speech rate and syntactic complexity similarly constrain cognitive processing in comprehension tasks (Wingfield et al., 2003), suggesting that duration effects may be comparable in magnitude to these well-established factors in speech processing. The performance decline pattern was non-linear: the sharp drop from 80s to 140s (Mean Difference = 0.12, *p* < .001) contrasted with the more gradual decline from 140s to 200s (Mean Difference = 0.10, *p* = .003). Expressed as relative declines, performance decreased by 43% between the first two durations but only 25% between the latter two. This pattern suggests a functional threshold around 80–140 s where note-decoding transitions from relying primarily on immediate working memory to requiring hierarchically structured external memory systems (i.e., well-organized notes).

This threshold aligns with working memory research on sustained information processing capacity (Cowan, 2001, 2022), which identifies similar temporal limits for maintaining and manipulating information without external support. The convergence between our empirically derived threshold and theoretical predictions from cognitive psychology suggests that the 100–140 s range represents a transition point where cognitive demands exceed novice interpreters’ available resources, triggering a qualitative shift in processing strategies. By isolating duration as an independent variable under controlled conditions, this finding extends previous CI research beyond global performance measures, providing phase-specific evidence of cognitive thresholds in the reformulation stage.

While our results align with the “resource competition” logic central to Gile’s (2009) Effort Model, they suggest two theoretical refinements. First, the non-linear decline pattern implies a duration-sensitive threshold effect rather than a simple linear trade-off among competing efforts. This suggests that Gile’s model, while capturing the competitive dynamics among Rem, Read, and P efforts, may benefit from incorporating threshold parameters that account for qualitative shifts in processing demands at key duration points. Second, the strengthening correlation between anxiety and performance at longer durations (80s: *r* = − .45; 200s: *r* = − .67) indicates that resource availability is jointly shaped by cognitive and affective factors. This interaction suggests that the “total available resources” in Gile’s formulation should be conceptualized as modulated by emotional states, particularly under extended temporal demands that exceed automatized processing capacity.

### Understanding the non-linear decline

Two mechanisms explain the observed non-linear performance patterns. First, temporal distance and cue sufficiency effects become more pronounced as duration increases. The temporal gap between encoding and decoding widens, increasing reliance on notes as external memory. The 80s→140s decline suggests a qualitative shift in which elliptical, personalized notes effective for shorter segments can no longer support efficient reconstruction without explicit macro-structure and cross-references. This finding supports Ahrens and Orlando’s (2022) conceptualization that “good notes” externalize the discourse scaffold rather than transcribe content, demonstrating that note-decoding effectiveness deteriorates disproportionately as duration increases.

Second, cognitive overload and affective factors intensify the decline pattern through interactive mechanisms. Longer segments encompass more propositional groupings and cross-sentential relations that require hierarchical organization in working memory. When notes fail to capture these relations through clear hierarchy, reconstruction costs increase non-linearly (Ahrens & Orlando, 2022; Mellinger, 2022), creating a compounded burden on Rem and Read that manifest as a steep decline around the middle duration. This cognitive burden is accompanied by increased anxiety, which rose with duration (ηp² = 0.35) and correlated more closely with performance at longer durations (80s: *r* = − .45; 200s: *r* = − .67). The strengthening anxiety-performance correlation suggests that extended durations exceed novice interpreters’ pattern recognition capabilities, forcing reliance on analytical strategies that are more vulnerable to anxiety disruption (Eysenck et al., 2007). This pattern aligns with recent findings by Gumul and Pérez-Luzardo Díaz (2025), who found that under high cognitive load, interpreters employ strategies primarily as coping mechanisms rather than for strategic enhancement, a distinction that becomes increasingly pronounced as task demands escalate.

Figure 3 shows a temporal dissociation in the trajectories of performance indicators (TR, OP, TRatio) and anxiety across the three duration conditions. While performance measures show their steepest decline between 80s and 140s (Cohen’s d = 1.15–1.24), anxiety shows its largest increase between 140s and 200s (Cohen’s d = 1.34). This pattern suggests that performance deterioration occurs earlier than peak anxiety, a finding with implications for training interventions targeting the 100–140 s threshold. The temporal lag between performance decline and peak anxiety indicates that cognitive overload, rather than anxiety per se, serves as the primary mechanism driving performance decline, with anxiety emerging as a secondary response to cognitive strain. Once elevated, anxiety can further impair retrieval efficiency, narrow attentional scope, and trigger conservative strategies (Eysenck et al., 2007), potentially creating a feedback loop that compounds decoding difficulties. This cascade effect, where initial cognitive overload triggers anxiety, which then amplifies cognitive constraints, suggests that interventions targeting the 100–140 s threshold should address both cognitive load management and anxiety regulation to prevent performance deterioration at extended durations.

### Individual differences and strategic resilience

Analysis of individual differences revealed insights about strategic resilience. While most participants (86.7%) showed monotonic decline, 13.3% exhibited non-monotonic patterns, with some maintaining high performance across durations. These individual differences align with research showing that performance variations stem primarily from knowledge structure and strategic differences rather than raw cognitive capacity (Feltovich et al., 2018).

Resilient performers showed smaller performance decline from 80s to 200s (23% vs. 57% for the group overall), suggesting that effective duration management strategies exist and can be taught through deliberate practice. These individuals likely developed protective factors such as structured note-taking systems, automated encoding strategies, and emotion regulation skills that stabilize performance under pressure. This pattern aligns with Ericsson and Kintsch’s (1995) theory of long-term working memory, which posits that expert performers develop domain-specific retrieval structures enabling rapid access to stored information. These individuals bypass typical working memory capacity constraints through better memory organization in their domain (Ericsson, 2018), allowing them to maintain performance even under extended temporal demands.

The mechanisms underlying this resilience require further investigation. Resilient performers may possess greater working memory capacity, more effective note-taking strategies, or stronger emotion regulation skills that buffer against duration-induced decline. Alternatively, they may have developed more efficient resource allocation strategies that remain effective across durations. Distinguishing between these mechanisms requires investigation of working memory capacity, note-taking strategies, and anxiety regulation.

### Positioning within existing literature

Our findings extend three research strands. First, within Gile’s (2009) Effort Model framework, we provide validation of the Read component by demonstrating measurable, duration-sensitive effects under controlled conditions. The performance decline supports the model’s resource competition logic while revealing a non-linear threshold effect, a steeper decline between 80s and 140s, followed by a more gradual decline from 140s to 200s. This pattern suggests that Read capacity exhibits a threshold beyond which performance degradation accelerates, a finding that refines our understanding of how resource competition operates in decoding.

Second, while previous cognitive load research focused on listening and note-taking (Dong & Li, 2020; Seeber, 2011, 2013), our study documents a duration-induced bottleneck at the decoding stage, completing the resource competition framework across all sequential phases of the CI process. By identifying decoding as a locus of duration effects, we extend CLT’s intrinsic/extraneous load distinction to retrieval and reconstruction processes. As duration increases, the intrinsic load of integrating noted and memorized information increases non-linearly, while extraneous load increases due to limited discourse scaffolding in notes. The non-linear performance pattern reflects this compounding of intrinsic and extraneous loads at the 80–140 s threshold.

Third, within note-taking scholarship (Chen, 2017; Dam, 2021; Ghiselli, 2022; Gillies, 2017; Mellinger, 2022), our data support conceptualizing notes as elements of a distributed cognitive system that extends beyond individual memory to encompass externalized, structured representations. In this framework, note-taking effectiveness depends on successful externalization of the discourse skeleton (the hierarchical structure of main ideas and their relationships), not merely content transcription. Our findings demonstrate that beyond the 80–140 s threshold, limited externalization of discourse structure leads to steep decoding costs. This threshold effect provides evidence that notes function as integral cognitive processing components rather than external storage devices (Chen, 2017; Zou & Guo, 2024). Interpreters who develop hierarchical note structures can maintain performance across extended durations by distributing cognitive processing across memory and notes.

### Pedagogical and assessment implications

Our findings suggest a duration progression for interpreter training. The threshold between 80s and 140s suggests interpreter training should address the transition from shorter to longer segments through a three-stage progression: (1) mastery of 60–90 s segments with basic note-taking techniques, (2) focused practice at 100–140 s on hierarchical discourse organization, and (3) training on 180s + segments. Training should emphasize targeted practice rather than accumulated experience. The resilient performers in our study demonstrate that effective strategies can be taught through exercises targeting the mechanisms underlying performance decline. These include hierarchical note layout that externalizes discourse structure, relational marking (cause, contrast, sequence), and Read + Rem practice under time pressure. These interventions address the discourse skeleton externalization that our findings identify as critical for maintaining performance across durations.

Formative assessment should incorporate transformation rate (TR) and transformation ratio (TRatio) as diagnostic metrics, paired with qualitative analysis of note structure. Instructors should evaluate whether notes capture hierarchical organization of main ideas, whether cues are sufficiently specific for retrieval, and whether cross-page references enable discourse continuity. This dual approach provides feedback on the mechanisms underlying performance decline. The strengthening anxiety-performance correlation with longer durations suggests that training should include extended segments (140s+) to familiarize students with the cognitive demands of longer tasks.

Given the large effect sizes observed (Cohen’s d = 1.15–1.34), duration should be treated as a difficulty parameter in assessment contexts. Programs should establish duration categories (≤ 90s short; 90–150 s medium; ≥180s long) and report duration alongside performance scores to enable fair benchmarking. Assessment protocols should recognize that 200s segments impose different cognitive demands than 80s segments and establish duration-specific benchmarks or weight performance by duration category. Assessment should also incorporate anxiety measures to distinguish anxiety-related decrements from cognitive capacity limitations. This distinction helps identify whether students require cognitive strategy training or anxiety management support.

### Limitations and future directions

This study has several limitations that should be acknowledged. First, the fixed presentation order (80s→140s→200s) makes it difficult to fully separate duration effects from order and fatigue effects. Although this ascending design reflects training progressions, performance changes may reflect practice effects, expectancy violations, or cumulative fatigue rather than duration per se. Our indirect checks—standardized inter-segment breaks, retrospective tiredness reports, and within-segment error comparisons—are suggestive but not conclusive. Future work should employ counterbalanced designs or use mixed-effects models to control for order effects while preserving statistical power. Second, our sample is limited to novice English-Chinese interpreters from a single institution with similar backgrounds. The unidirectional L2→L1 design may not reflect bidirectional interpreting demands in professional settings. The gender imbalance (96.7% female) constrains representativeness. Cross-linguistic studies with different language pairs, samples from multiple institutions, and more balanced demographics are needed. Third, retrospective interviews may introduce memory biases and social desirability effects and may not capture cognitive dynamics in real time. While this approach avoided disrupting performance, the discrepancies between formal difficulty ratings and interview data suggest that single-dimension scales inadequately capture interpreting difficulty’s multidimensional nature.

Building on these limitations, several research directions emerge. First is clarifying whether the 100–140 s threshold is universal or context-specific. Does this threshold hold across language pairs, directionalities (L1→L2 vs. L2→L1), proficiency levels, and cultural contexts? If the threshold is universal, it suggests a cognitive constraint; if context-specific, it may reflect trainable strategies or language-specific factors. Answering this question requires cross-linguistic studies and longitudinal research tracking skill development from novice to expert levels. Second is identifying what distinguishes resilient performers: superior working memory capacity, more effective note-taking strategies, or better emotion regulation. Studies incorporating working memory assessments and analysis of note-taking patterns could identify cognitive predictors of resilience. Such findings would inform training interventions. Third, intervention studies could test whether training reduces duration-induced decline. For example, does training in hierarchical note-structuring mitigate performance decline at longer durations? Does anxiety management training weaken the anxiety-performance correlation? These questions move from documenting the problem to testing solutions. Finally, future research should employ counterbalanced designs to isolate duration effects from order and fatigue, while incorporating real-time process measures (e.g., eye-tracking, think-aloud protocols) to complement retrospective self-reports.

## Conclusion

This study demonstrates that source language duration significantly affects note-decoding effectiveness in consecutive interpreting. We tested novice interpreters with three durations (80s, 140s, 200s) while controlling for speech rate and content, and found that performance declined non-linearly: a sharp drop from 80s to 140s, then a more gradual drop to 200s. At the same time, anxiety-performance correlations strengthened at longer durations. These results support Gile’s Effort Model and clarify when capacity constraints become problematic in Phase Two of consecutive interpreting.

Three key contributions emerge from this work. First, we isolate duration as a difficulty parameter independent of speech rate and content complexity. Second, we identify cognitive overload as the main mechanism driving performance decline, with anxiety intensifying at longer durations. Notably, this pattern suggests that performance deterioration may precede anxiety responses rather than anxiety directly causing decline—a distinction relevant for training design. Third, we document individual differences in duration resilience. Some interpreters maintain stable performance across durations, indicating that effective strategies exist and can be taught.

For training and assessment, several implications follow. Programs should work explicitly on the 100–140 s range through note-structuring exercises and coordinated memory-decoding practice. Assessment should use duration-specific standards and report duration with scores, since longer segments create qualitative different cognitive demands. Importantly, since some interpreters perform well across durations, training should focus on teaching the specific skills they use: how to organize discourse clearly in notes, mark relationships explicitly, and manage emotion under pressure.

This study connects cognitive theory to empirical findings about what happens when interpreters work with longer segments. By identifying where note-decoding breaks down and why, it provides a foundation for improving training and assessment practices. As professional interpreting often involves extended discourse, developing the ability to handle longer segments will remain important for interpreter competence.

## Data Availability

The datasets used in this study are available from the corresponding author upon reasonable request.
